# Modular Endoprostheses for Nonneoplastic Conditions: Midterm Complications and Survival

**DOI:** 10.1155/2016/2606521

**Published:** 2016-12-05

**Authors:** Marco De Gori, Guido Scoccianti, Filippo Frenos, Leonardo Bettini, Filippo Familiari, Giorgio Gasparini, Giovanni Beltrami, Pierluigi Cuomo, Pietro De Biase, Rodolfo Capanna

**Affiliations:** ^1^Department of Orthopaedic and Trauma Surgery, “Mater Domini” University Hospital, “Magna Graecia” University of Catanzaro, Catanzaro, Italy; ^2^Department of Orthopaedic Oncology and Reconstructive Surgery, Careggi University Hospital, University of Florence, Florence, Italy; ^3^Department of Orthopaedic and Trauma Surgery, Careggi University Hospital, University of Florence, Florence, Italy; ^4^Department of Orthopaedic and Trauma Surgery, University of Pisa, Pisa, Italy

## Abstract

The use of modular endoprostheses is a viable option to manage both tumor resection and severe bone loss due to nonneoplastic conditions such as fracture sequelae, failed osteoarticular grafts, arthroplasty revisions, and periprosthetic fractures. We sought to investigate both midterm complications and failures occurred in 87 patients who underwent a megaprosthetic reconstruction in a nonneoplastic setting. After a mean follow-up of 58 (1–167) months, overall failure-free survival was 91.5% at 1 year, 80% at 2 years, 71.6% at 5 years, and 69.1% at 5 and 10 years. There was no significant difference in the survival rate according to the diagnosis at the index procedure (*p* = 0.921), nor to the reconstruction site (*p* = 0.402). The use of megaprostheses in a postneoplastic setting did not affect survival rate in comparison with endoprosthetic reconstruction of pure nonneoplastic conditions (*p* = 0.851). Perimegaprosthetic infection was the leading complication, occurring in 10 (11.5%) patients and implying a megaprosthetic revision in all but one case. Physicians should consider these results when discussing with patients desired outcomes of endoprosthetic reconstructions of a nonneoplastic disease.

## 1. Introduction

Modular endoprosthesis is a well-established reconstructive device in orthopaedic oncology to manage wide bone resections due to the relatively simple and quick intraoperative assembly and immediate mechanical stability, allowing early weight bearing and functional recovery [1]. The outstanding advances in both megaprostheses' materials and designs allowed progressively expanding the indications for their use in the treatment of nonneoplastic conditions, as in the case of acute trauma in severe bone loss and poor bone quality, posttraumatic failures represented by complex nonunions and critical size bone defects, major bone loss in prosthetic revision, periprosthetic fractures with component loosening, and poor bone stock [2–14].

Two recent systematic reviews [6, 7] focused on the widespread use of modular endoprostheses for nonneoplastic conditions. Authors found an overall midterm survival rate of 76% for proximal femoral prostheses [6] and 83% for distal femoral prostheses [7]. Notably, posttraumatic, infective, and periprosthetic conditions have peculiar characteristics that are very different to those of oncologic patients. Patient's general condition and comorbidities, soft-tissue status, lesion characteristics, muscle depletion, previous surgeries, presence of adhesions, and any previous sepsis all are factors that must be carefully considered when using megaprosthesis in such cases [5]. In this light, to maximize functional improvement and minimize the risk of postoperative complications, we do believe that endoprosthetic reconstructions of a nonneoplastic disease should be performed in high-volume centers to treat well selected patients after a careful consideration of both surgical alternatives and desired outcomes.

In this work, we aimed to evaluate our experience in the use of modular endoprostheses for the treatment of nonneoplastic conditions. Particularly, we sought to investigate both complications and failures occurred in our patients.

## 2. Materials and Methods

### 2.1. Study Population

The study was approved by the local ethical committee, and the research was performed in compliance with the Helsinki Declaration. We reviewed data from consecutive patients who have undergone a modular endoprosthetic replacement at our institution from January 2001 to December 2014. Patients who received a megaprosthetic implant for the treatment of a nonneoplastic or postneoplastic (i.e., the treatment of a failed oncologic surgery in a recurrence-free patient) condition are included in this retrospective study.

In the study period, 509 modular endoprostheses of the lower limb were implanted in 509 patients. Of them, 87 were used to manage nonneoplastic or postneoplastic conditions of severe bone loss and were included in the study. There were 54 (62.1%) females and 33 (37.9%) males, averaging 61 (18–93) years at the index procedure.

Indications for the use of the megaprosthesis were the management of fracture sequelae in 34 (39.1%) patients, total hip arthroplasty revision in 23 (26.4%) patients (due to aseptic loosening in 15 cases, periprosthetic joint infection in 7 cases, and instability in 1 cases), a total knee arthroplasty revision in 14 (16.1%) patients (due to periprosthetic joint infection in 8 cases, aseptic loosening in 5 cases, and prosthesis disassembly in 1 case), the management of sequelae of a failed osteoarticular graft in 7 (8%) patients, the management of a periprosthetic fracture in 4 (4.6%) patients, a megaprosthesis revision in 4 (4.6%) patients, and a combined total hip and knee arthroplasty revision (due to aseptic loosening) in 1 (1.1%) patient. Overall, in 25 (28.7%) patients' clinical history a previous surgical site infection has been detected. Twenty-one (24.1%) patients underwent megaprosthetic reconstruction in postneoplastic conditions; of them, 5 patients previously underwent radiation therapy, 4 patients previously underwent chemotherapy, and 2 patients previously underwent a combined chemotherapy and radiation therapy.

### 2.2. Surgical Technique

All surgical procedures were performed under general anesthesia. The Megasystem-C® (Waldemar LINK® GmbH & Co. KG, Hamburg, FRG) modular system was used in all cases. In detail, 40 (46%) proximal femoral endoprostheses, 26 (29.9%) distal femoral endoprostheses, 9 (10.3%) proximal tibial endoprostheses, 8 (9.2%) knee arthrodesis endoprostheses, and 4 (4.6%) total femoral endoprostheses were implanted.

A conventional megaprosthesis was used in all but 3 patients that received a proximal tibial allograft-prosthesis composite. Among patients complaining of a previous surgical site infection, 12 of them received a silver-coated endoprosthesis trying to minimize the risk of postoperative perimegaprosthetic infection [15]. For endoprosthetic reconstructions of the knee, reconstruction of the extensor mechanism was accomplished by suturing the patellar tendon on the tibial component, which provides a fixation cage on its proximal part. A rotating hinge mechanism was used in all cases, and resurfacing of the patella was not performed in any case. In proximal femoral procedures, reconstruction of the abductor mechanism was accomplished by suturing the vastus lateralis and gluteus medius on the prosthesis, which provides holes on its proximal part.

The implant system we used provides either cemented or cementless stems. Cemented fixation of the stem was used in our series in 60% of cases. In detail, we preferred to use cemented stems in irradiated bone, in old patients with osteoporotic bone, and when the shape of the medullary canal did not allow an adequate press-fit fixation [1].

All patients received a 6-week pharmacological subcutaneous thromboembolism prevention therapy with low-molecular-weight heparin. Standard antibiotic proxylaxis regimen consisted of a one-week pharmacological venous administration of vancomycin-containing prophylaxis followed by a one-week oral administration of *β*-lactams; this regimen was modified in specific cases as a previous surgical site infection, renal insufficiency, or allergy.

### 2.3. Patient Evaluation

A complete clinical history was obtained from all patients. Initial and follow-up data were extracted from our medical records relating to follow-up evaluations. Patients with insufficient data in our database were recalled and evaluated. Specifically, patients were reviewed for both complications and failure of their megaprosthetic implant. Complications were classified according to the system by Henderson et al. [16], as previously modified for its use in nonneoplastic conditions [6], into soft-tissue complications (type 1), aseptic loosening (type 2), structural complications (type 3), and perimegaprosthetic infections (type 4). Failed reconstructions were defined as those having required complete revision of the endoprosthesis, unplanned revision of a failed portion of the endoprosthesis, fixation of a periprosthetic fracture, soft-tissue reconstruction to restore joint stability, endoprosthetic removal without revision, and amputation [16]. The mean follow-up was 58 (1–167) months.

### 2.4. Statistical Analysis

The mean and range were reported for the continuous variables, whereas counts and percents described the categorical variables. Survival curves were established with the Kaplan-Meier method [17], and the difference in cumulative survival between groups of patients was assessed with the Mantel-Cox log-rank test. The Cox regression analysis was used to test the effect of possible covariates.

The IBM SPSS Statistics 21.0.0.1 software (IBM Corp., Armonk, NY, USA) was used for the database construction and the statistical analysis. 2-tailed *p* < 0.05 was considered significant.

## 3. Results

### 3.1. Soft-Tissue Complications

Eight cases (9.2%) of dislocation were recorded among the 44 proximal or total femoral endoprostheses, occurring 12 (1–40) months after the index procedure. In all but 1 case, a bipolar head has been used; 1 constrained cup has been furtherly used. Overall, 3 dislocations were recurrent. A closed reduction was effectively performed in 4 patients, whereas 4 patients underwent an acetabular component revision (with retentive cups having been used in 2 cases) with or without an associated femoral component revision.

One patient treated with a proximal tibial endoprosthesis experienced an extensor mechanism failure 52 months postoperatively, and an allograft reconstruction was performed. A mobilization of the fixation cage for the patellar tendon occurred in 2 cases 12 and 52 months after the index procedure; as no deficit of the extensor mechanism occurred, the cage was removed in both cases.

With failures related to soft-tissue complications considered as endpoint, endoprostheses survival was 96.5% at 1 year, 95.1% at 2 years, and 93% at 5 and 10 years.

### 3.2. Aseptic Loosening

Overall, aseptic loosening occurred in 2 (2.3%) cases 23 months postoperatively. Specifically, one patient who underwent a distal femoral replacement exhibited a mobilization of the cemented femoral stem, thus requiring a megaprosthetic revision and the implant of a total femoral endoprosthesis. One patient experienced a posttraumatic mobilization of the acetabular component of a proximal femoral endoprosthesis, and a revision of the component was performed.

With failures related to aseptic loosening considered as endpoint, endoprostheses survival was 100% at 1 years and 97.1% at 2 at 5 and 10 years.

### 3.3. Structural Complications

Globally, 6 (6.9%) structural complications were noted. Specifically, 4 periprosthetic fractures of the femur (3) and tibia (1) meanly occurred 41 (23–59) months after the index procedure. One fracture healed with a long leg cast immobilization, one further case required an internal fixation and bone graft implant, and a megaprosthetic revision was performed in the remnant 2 cases. A megaprosthetic disassembly occurred 22 months postoperatively due to a fracture of one of the Morse tapers connecting two contiguous modules: a revision of the involved modules was then performed. A fracture of both the ceramic component and polyethylene insert of a proximal femoral megaprosthesis occurred 24 months postoperatively: a megaprosthetic revision was then performed.

With failures related to structural complications considered as endpoint, endoprostheses survival was 100% at 1 year, 95.6% at 2 years, and 90.4% at 5 and 10 years.

### 3.4. Perimegaprosthetic Infections

Overall, infection occurred in 10 (11.5%) patients (in detail, 5 proximal femoral endoprostheses, 3 distal femoral endoprostheses, 1 proximal tibial endoprosthesis, and 1 total femoral endoprosthesis) 23 (1–63) months after the index procedure. Of them, 3 patients had history of a previous surgical site infection, 3 patients received a silver-coated endoprosthesis, and 2 patients previously underwent radiation therapy. In all but one case a megaprosthetic revision was performed: in detail, one patient received a suppressive antibiotic therapy on the basis of his medical status and contraindication to surgery, and he died from comorbidities being the infection still persistent. Of the 9 performed surgeries, there were 6 two-stage revisions (in one case the second stage was not performed due to the patient's death) and 3 one-stage revisions. Finally, infection still persisted in 2 patients that underwent a two-stage revision, and a hip disarticulation was then performed.

With failures related to perimegaprosthetic infections considered as endpoint, endoprostheses survival was 95.1% at 1 year, 90.7% at 2 years, 89% at 5 years, and 86.3% at 10 years. There was no significant difference in the survival rate according to the reconstruction site (*p* = 0.987), nor to the history of a previous surgical site infection (*p* = 0.238).

### 3.5. Overall Survival Analysis

With any implant failure considered as endpoint, whichever first occurred, overall endoprostheses survival was 91.5% at 1 year, 80% at 2 years, 71.6% at 5 years, and 69.1% at 5 and 10 years. There was no significant difference in the survival rate according to the diagnosis at the index procedure (*p* = 0.921), nor to the reconstruction site (*p* = 0.402).

The use of megaprostheses in a postneoplastic setting did not affect survival rate in comparison with endoprosthetic reconstruction of nonneoplastic conditions (*p* = 0.851).

## 4. Discussion

The development of megaprostheses for large resections has provided important options to orthopaedic oncologist surgeons for the replacement of skeletal segments. Megaprostheses are currently gaining momentum, at least in high-volume centers, as a useful and effective reconstructive strategy for severe bone loss following nonneoplastic conditions, such as a failed joint replacement or fracture, complex periprosthetic fractures, and severe instabilities of distal femoral prostheses. Though promising results have been published [2–14], the use of megaprostheses in such cases should be considered as a limb salvage option in carefully selected patients, when other surgical options are unfeasible [6].

Implant survival is still the main concern that may limit the routine use of endoprostheses to manage nonneoplastic conditions. We found an overall failure-free survival was 91.5% at 1 year, 80% at 2 years, 71.6% at 5 years, and 69.1% at 5 and 10 years, without significant difference in the survival rate according to the diagnosis at the index procedure nor to the reconstruction site (Table 1). Our results slightly differ from those of the previously published literature: Berend and Lombardi [18] found encouraging results with an overall reoperation-free survival of 97% at 1 year, 95% at 2 years, and 83% at 5 years after distal femoral replacement. On the contrary the systematic reviews by Korim et al. [6, 7] pointed out a mean failure rate of 76% at 3.8 years for proximal femoral prostheses [6] and 83% at 3.3 years for distal femoral prostheses [7]. We may explain such discrepancies by assuming that our survival analysis was carried out considering failures according to Henderson et al. [16] rather than overall reoperations as endpoints.

Periprosthetic joint infection remains one of the most challenging complications following joint replacement and a leading cause of early implant failure [19]. Whilst overall infection rate is relatively low, being approximately 1% following hip and knee arthroplasties [20], it might dramatically increase in the presence of certain risk factors, as in the case of patients' poor health status, extensive soft-tissue dissection, long operating times, and the need of multiple blood transfusions [21–23]. In this light, a deep infection may be a devastating complication following megaprosthetic replacement, posing a high risk for repeated surgical procedures, poor functional outcome, and failed limb salvage. Overall, a recent systematic review [24] has reported a mean rate of perimegaprosthetic infection of 10% following tumor resection. In the current series, an infection rate of 11.5% in nonneoplastic conditions has been observed, thus being in agreement with previous findings of a mean rate of 7.6% for proximal femoral prostheses [6] and 15% for distal femoral prostheses [7].

Several risk factors for perimegaprosthetic infections have been previously identified. Among them, a medical history complaining of a previous surgical site infection has been previously advocated as a leading risk factor for reinfection after tumor resection and endoprosthetic reconstruction [25, 26]. Data of the current study contrasts with those of the previous published literature, as we detected that the history of a previous surgical site infection did not affect survival rate. We may explain this discrepancy by assuming that the use of silver-coated megaprostheses in such critical patients might have minimized the risk of perimegaprosthetic infection [15].

We detected 8 cases of dislocation, being the 18% of proximal and total femoral endoprostheses of our series; a revision procedure was performed in 4 (4.6%) patients. Our results are in agreement with those of the systematic review by Korim et al. [6], reporting a rate of 15.7% at a mean follow-up of 45 months, thus dislocation being most common complication of proximal femoral prostheses for nonneoplastic conditions. Of interest, a higher midterm rate of 37.5% has been previously reported when using proximal femoral endoprostheses as revision arthroplasties [13]. Conversely, dislocation rate of modular endoprostheses for tumor reconstruction is globally lower. In their retrospective review of 2174 patients, Henderson et al. [16] detected an overall rate of soft-tissue complications (i.e., including dislocation) of 5.2% in primary proximal femoral prostheses. Moreover, we previously detected a dislocation rate of 4.3% in proximal femoral resections of 200 patients [1]. While conjectural, we may assume that such a discrepancy between dislocation rates is related to the systematic acetabular replacement in nonneoplastic conditions, whereas tumor resection often benefits from a cephalic megaprosthesis.

Both structural failures and aseptic loosening represent minor complications in the current series, with low rates being consistent with those we previously detected after tumor resection [1]. Of interest, aseptic loosening of megaprostheses in the treatment of nonneoplastic diseases has been previously detected with rates ranging from 0% to 9.5% [2, 3, 8–10, 13].

Main limitations of this study include the small sample size and lack of a control group with a different endoprosthetic reconstruction system. The average length of follow-up (i.e., 58 months) is still inadequate to draw definite conclusions on the implant longevity and survivorship, posing a certain risk to underestimate the actual failure rate. As a further limitation, we did not perform a postoperative evaluation of functionality and quality of life of the patients [18, 27]. Limits and potential biases arising from the retrospective design should be finally considered.

## 5. Conclusions

Our results support the use of modular endoprostheses as a solution to manage complex nonneoplastic diseases. Though perimegaprothetic infection still remains the leading complication, an overall survival rate of 69.1% at 10 years, without significant difference in the survival rate according to the diagnosis at the index procedure nor to the reconstruction site, represents a promising result in such a complex surgical procedure. Indeed, in cases of severe bone loss associated with a failed joint replacement or fracture, a megaprosthetic reconstruction may be the sole effective procedure that can be performed.

## Figures and Tables

**Table 1:** Overview of complications according to the surgical site.

	Proximal endoprostheses: PF and TF endoprostheses, *n* = 44	Distal endoprostheses: DF, KA, and PT endoprostheses, *n* = 43
Type 1: soft-tissue complications, *n* = 11	8	3
Type 2: aseptic loosening, *n* = 2	1	1
Type 3: structural complications, *n* = 6	4	2
Type 4: perimegaprosthetic infections, *n* = 10	6	4

PF: proximal femoral.

TF: total femoral.

DF: distal femoral.

KA: knee arthrodesis.

PT: proximal tibia.
